# Maternal body mass index moderates antenatal depression effects on infant birthweight

**DOI:** 10.1038/s41598-019-42360-1

**Published:** 2019-04-17

**Authors:** Heidrun Petursdottir Maack, Alkistis Skalkidou, Anna Sjöholm, Karin Eurenius-Orre, Ajlana Mulic-Lutvica, Anna-Karin Wikström, Inger Sundström Poromaa

**Affiliations:** Department of Women’s and Children’s Health, Uppsala University, 751 85 Uppsala, Sweden

**Keywords:** Depression, Pregnancy outcome

## Abstract

Obesity and depression are two common medical problems that pregnant women present with in antenatal care. Overweight and obesity at the beginning of the pregnancy, and excessive weight gain during pregnancy, are independent explanatory variables for fetal birthweight and independent risk factors for giving birth to a large for gestational age (LGA) infant. However, the effect of co-morbid depression has received little attention. This study set out to investigate if maternal body mass index (BMI) in early pregnancy moderates antenatal depression effects on infant birthweight. 3965 pregnant women participated in this longitudinal cohort study, where cases (n = 178) had Edinburgh Postnatal Depression Scale (EPDS) score ≥ 17 in gestational week 17 or 32, and remaining women (n = 3787) were used as controls. The influence of maternal BMI and antenatal depressive symptoms on standardized birthweight was evaluated by analysis of covariance, with adjustment for relevant confounders. Depressed women with BMI 25.0 kg/m^2^ or more gave birth to infants with significantly greater standardized birthweight than non-depressed overweight women, whereas the opposite pattern was noted in normal weight women (BMI by antenatal depressive symptoms interaction; F(1,3839) = 6.32; p = 0.012. The increased birthweight in women with co-prevalent overweight and depressive symptoms was not explained by increased weight gain during the pregnancy. Maternal BMI at the beginning of pregnancy seems to influence the association between antenatal depressive symptoms and infant birthweight, but in opposite directions depending on whether the pregnant women is normal weight or overweight. Further studies are needed to confirm our finding.

## Introduction

Obesity and depression are two common medical problems that pregnant women often present with in antenatal care. In the general population, these two conditions are often encountered in the same individuals, with approximately one in four obese women also being depressed^1^. In pregnancy, overweight and obesity are associated with a number of adverse obstetric and perinatal outcomes including preterm birth, preeclampsia and gestational diabetes^2,3^. In addition, overweight and obesity, as well as excessive weight gain during pregnancy, are independent explanatory variables for fetal birthweight and independent risk factors for giving birth to large for gestational age (LGA) infants^4,5^.

Depression during pregnancy, on the other hand, has also been associated with a number of adverse neonatal outcomes, including preterm birth and low birthweight^6^. Similarly, antenatal anxiety disorders and anxiety symptoms seem to increase the risk of spontaneous preterm birth and low birthweight^7^, and this finding has also been demonstrated in women with comorbid anxiety and depression^8–10^. Some studies also suggest that antenatal anxiety confers a stronger risk for low birthweight than antenatal depression^11,12^. These complications may partly be explained by the fact that antenatal mental health problems are associated with a number of characteristics that increases the risk for low birthweight such as low socioeconomic status, smoking, and drug abuse^13–15^. Indeed, a recent meta-analysis pointed to a multifaceted picture, where socioeconomic status also influenced the relationship between antenatal depression and birthweight^6^. At the same time, an increasing number of reports suggest that obesity is common among women with antenatal depression^16,17^, and two reports have suggested that antenatal depression also may lead to increased birthweight^18,19^.

Thus, in light of the increasing prevalence of overweight and obesity in the pregnant population, and in view of the conflicting and limited information^6,20^, this study aimed to investigate if maternal body mass index (BMI) in early pregnancy moderates antenatal depression effects on infant birthweight. A secondary aim was to assess if the relationship between antenatal depressive symptoms and birthweight is mediated by gestational weight gain.

## Material and Methods

### Primary study population

The BASIC project (Biology, Affect, Stress, Imaging, Cognition in the Puerperium) is a population-based, longitudinal study of psychological well-being during pregnancy and the postpartum period in Uppsala County, Sweden. The invitation to the BASIC study is sent out together with the invitation for the ultrasound examination around 17 weeks of gestation, and women included in this nested case-control study were included between September 2009 and January 2016. Exclusion criteria for the BASIC study are (1) inability to adequately communicate in Swedish, (2) women with confidential personal data, (3) women with pathologic pregnancies as diagnosed by routine ultrasound (miscarriages and congenital malformations), and (4) women younger than 18 years. Participation rate in the BASIC study is approximately 22%. By June 2016, 4623 participants in the BASIC project had given birth at Uppsala University Hospital. Of these, we excluded 67 twin pregnancies, 39 cases with missing infant birthweight, 297 (6.4%) women with no information on BMI at the first antenatal booking, and 255 women who entered the study after gestational week 32. Thus, the final sample size consisted of 3965 pregnant women. The proportion of women with missing information on BMI was similar to the overall Swedish population, Table 1.

**Table 1:** Birth statistics in the primary study population, in Uppsala County, and in the general population of Sweden, only singleton pregnancies. Data expressed as mean ± SD or n (%).

Variable	Primary study population 2009–2016 (n = 3965)	Uppsala County 2009–2014 (n = 23 352)	Sweden^a^ 2009–2014 (n = 625 118)
Maternal age, years	31.4 ± 4.6	30.4 ± 5.2	30.3 ± 5.3
Primiparous, n (%)	1 865 (47.0)	10 270 (44.0)	275 909 (44.1)
Country of origin			
Nordic countries	3 611 (91.1)	18 757 (80.3)	479 067 (76.6)
Europe	187 (4.7)	719 (3.1)	21 457 (3.4)
Outside Europe	129 (3.3)	3 876 (16.6)	124 594 (19.9)
Education ≤ 12 years	823 (21.3)	10 910 (46.7)	303 371 (48.5)
Smoking	99 (2.5)	1 053 (5.2)	37 078 (6.1)
Maternal BMI, kg/m^2^	24.3 ± 4.2	24.8 ± 4.7	24.7 ± 4.7
Gestational length, days	279 ± 12	277 ± 15	279 ± 13
Birthweight	3597 ± 536	3553 ± 595	3543 ± 552
Small for gestational age, n (%)	30 (0.8)	443 (1.9)	13 862 (2.2)
Large for gestational age, n (%)	167 (4.2)	1 097 (4.7)	21 744 (3.5)

^a^Sweden, except Uppsala County.

Percentages given in relation to available information. Missing cases most commonly found for smoking (3.4%) and BMI (6.7%).

The study participants completed web-based, self-administrated structured questionnaires containing questions on demographic variables, smoking, prior psychiatric history, ongoing medication, and the Swedish validated version of the Edinburgh Postnatal Depression Scale (EPDS) in gestational weeks 17 and 32.

Information on maternal BMI and maternal weight gain was obtained from the standardized antenatal medical records. Weight measurements are taken at first antenatal booking (gestational week 12), in gestational week 32, and in gestational week 36. Weight gain in gestational week 32 was defined as difference between weight in gestational week 32 and weight at first antenatal booking, and this information was available in 3731 (94.1%) women. Weight gain in gestational week 36 was available in 2252 (56.8%) women.

Women were considered to suffer from antenatal depression if they reported EPDS scores ≥ 17, either in gestational week 17 or in gestational week 32. The EPDS is an internationally used 10-item self-reported questionnaire, designed to identify depressive symptoms in the peripartum period^21^. While the sensitivity of the EPDS is relatively low, between 0.47–0.71, the specificity as regards depressive disorders is excellent (pooled specificity 0.94–0.98)^22^. The chosen cut-off was based on a recent report from the Postpartum Depression: Action Towards Causes and Treatment (PACT) Consortium, suggesting EPDS scores ≥ 17 being indicative of moderate to severe depression^23^. Further, a previous meta-analysis on neonatal outcomes in women with antenatal depression suggested that the relationship between birthweight and depression was stronger in women with a clinical diagnosis of depression^6^.

Information on maternal clinical variables, pregnancy complications, and perinatal outcomes was derived from the standardized antenatal, obstetric, and pediatric medical records. Obstetric diagnoses according to the International Classification of Diseases 10 (ICD 10) are recorded in the obstetric medical records.

### Ethical approval

All participating women give written informed consent, and the BASIC study has been approved by the Independent Ethical Review Board of Uppsala, Sweden (Dnr 2009/171, approval July 1, 2009), and is conducted in accordance with the declaration of Helsinki.

### Outcome

Standardized birthweight scores (SDS) according to the gestational age and sex-specific Swedish birthweight curves^24^ were calculated and used in the analyses. Small for gestational age (SGA) and large for gestational age (LGA) were defined as a birthweight of more than two standard deviations below or above the mean weight for gestational age according to the national reference curve, respectively^24^.

### Comparison with the general population

The BASIC project, which is used for this nested case-control study, has a relatively low response rate (22%), and is thus susceptible to selection bias. For that reason, we wanted to present the prevalence of maternal characteristics, common obstetric complications, and the outcomes of interest (birthweight and LGA) in the primary study population, in the entire population of women giving birth in Uppsala County, and in the general Swedish population.

For this part, we used information from the Swedish Medical Birth Register, which is hosted by The Swedish National Board of Health and Welfare. In addition, information on educational level and origin of birth was provided by Statistics Sweden, with data from the Education Register and the Register of the Total Population, respectively. Individual record linkage between the registries was possible by use of unique personal registration numbers, which are assigned to Swedish residents at birth or upon immigration.

The Medical Birth Register contains data on 98% of all births in Sweden and includes prospectively collected data from the standardized antenatal, obstetric, and pediatric medical records, including reproductive history, smoking status, complications that occur during pregnancy, delivery, and the neonatal period. Complications during pregnancy and delivery are classified according to ICD 10, as noted by the responsible obstetrician at discharge from the hospital. For comparison with the BASIC study population, all mothers with singleton pregnancies, giving birth between January 1, 2009 and December 31, 2014, in Uppsala County (n = 23352) and remaining Sweden (n = 625118) were included in the two control study populations.

### Statistics

Comparisons between depressed and non-depressed women in the BASIC cohort were performed by independent t-tests and Chi-square tests. Correlations were performed by Spearman Rank correlation, as self-rated depression scores failed to meet the assumption of normal distribution.

The influence of maternal BMI and antenatal depression on standardized birthweight (continuous, and large for gestational age (yes/no)) was evaluated by analysis of covariance (ANCOVA) or logistic regression. In both analyses BMI (categorized as BMI < 25.0 kg/m^2^ vs. BMI ≥ 25.0 kg/m^2^) and depression status (case vs. control) were entered as fixed factors, evaluating the main effects of BMI and depressive symptoms as well as the interaction between these two factors. The BMI cut-off was chosen to capture overweight and obesity, and was chosen to yield relatively comparable group sizes. The ANCOVA and logistic regression model were adjusted for age (continuous), parity (nulliparous vs. parous), maternal height (continuous), maternal education (more or less than 12 years of education), maternal origin of birth (Nordic vs. non-Nordic), and smoking (yes/no), and in a second step also with BMI as a continuous variable. These covariates were chosen based on the findings in the bivariate analyses, whereas smoking and parity were forced into the model based on previous literature^13^.

Follow-up linear regression models were performed using dummy variables corresponding to (1) normal weight non-depressed women (reference) (2) normal weight women with depressive symptoms, (3) non-depressed overweight women, and (4) overweight women with depressive symptoms. This regression model was performed in two steps; model 1 incorporating potential confounders as described above, and model 2 incorporating weight gain in gestational week 32 as a potential mediator.

## Results

### Study population

While the BASIC study population differed from Uppsala County and remaining Sweden in some aspects, most notably the higher educational level and lower rate of women with non-Nordic origin, maternal BMI, birthweight, and prevalence of LGA infants were similar to the general population, Table 1.

Overall, 178 (4.5%) women were classified as depressed, whereas remaining women were used as controls (n = 3787). Women who were classified as depressed were younger, significantly less often cohabiting, had a lower educational level, and were less often of Nordic origin, Table 2. They had a similar BMI distribution, and had similar rates of common pre-pregnancy conditions and obstetric complications as the controls. Further, women with antenatal depressive symptoms gave birth to offspring with lower birthweight; however, the standardized birthweight did not differ from the controls, Table 2.

**Table 2:** Demographic data and clinical variables in depressed and non-depressed women in the primary study population. Data expressed as mean ± SD or n (%).

Variable		Depressed women n = 178	Non-depressed women n = 3787	*P*
Age, years		30.6 ± 5.0	31.5 ± 4.5	0.018
BMI, kg/m^2^	<18.5	2 (1.1)	66 (1.7)	0.5
	18.5–24.99	117 (65.7)	2485 (65.6)	
	25–29.99	41 (23.0)	859 (22.7)	
	30–34.99	9 (5.1)	265 (7.0)	
	≥35	9 (5.1)	112 (3.0)	
Smoking, n (%)		9 (5.1)	90 (2.4)	0.025
Non-Nordic origin, n (%)		25 (14.2)	291 (7.8)	0.002
Education ≤ 12 years, n (%)		53 (30.3)	770 (20.9)	0.003
Single mothers		10 (5.6)	41 (1.1)	<0.001
First pregnancy, n (%)		75 (42.1)	1790 (47.3)	0.2
Pregnancy complications	Gestational hypertension, n (%)	4 (2.2)	152 (4.0)	0.3
	Gestational diabetes, n (%)	0	15 (0.4)	1.0
	Preeclampsia, n (%)	4 (2.2)	120 (3.2)	0.7
Gestational length (days)		276 ± 14	279 ± 11	0.001
Birthweight (g)		3509 ± 600	3600 ± 534	0.028
Birthweight (SDS)		0.31 ± 1.00	0.31 ± 0.96	0.9
Small for gestational age		0	30 (0.8)	0.3
Large for gestational age		11 (6.2)	156 (4.1)	0.2

Missing cases present in 1.0–3.8% of cases, percentages given in relation to available responses. Depressed women are defined as women with EPDS score ≥ 17 in either gestational week 17 or 32. BMI = Body Mass Index. Statistical analyses by independent t-tests or Chi-square tests.

### Infant birthweight in relation to covariates

Standardized birthweight was in significant positive correlation with first trimester BMI, maternal height, gestational weight gain, and self-rated depression scores in gestational week 17, Table 3. Standardized birthweight was also significantly associated with parity, non-Nordic origin, and educational level, Supplementary Table 1.

**Table 3:** Spearman Rank correlations between standardized birthweight and continuous variables in the primary study population.

Variable	n (%)	Birth-weight (SDS)	EPDS gw 32	EPDS gw 17	Weight gain at term	Weight gain gw 32	BMI	Maternal height
Age	3965 (100)	0.02	−0.09^c^	−0.11^c^	−0.11^c^	−0.05^b^	0.03	0.04
Maternal height	3965 (100)	0.20^c^	−0.04^b^	−0.03	0.07^b^	0.08^c^	−0.07^c^	
BMI	3965 (100)	0.17^c^	0.06^c^	0.06^c^	−0.04^a^	−0.09^c^		
Weight gain gw 32	3731 (94.1)	0.20^c^	0.04^a^	0.04^b^	0.86^c^			
Weight gain at term	2252 (56.8)	0.21^c^	0.06^b^	0.07^b^				
EPDS gw 17	3866 (97.5)	0.05^b^	0.67^c^					
EPDS gw 32	3793 (95.7)	0.03						

^a^p < 0.05, ^b^p < 0.01, ^c^p < 0.001, gw = gestational week.

### Infant birthweight in relation to depressive symptoms and BMI

**Figure 1:**
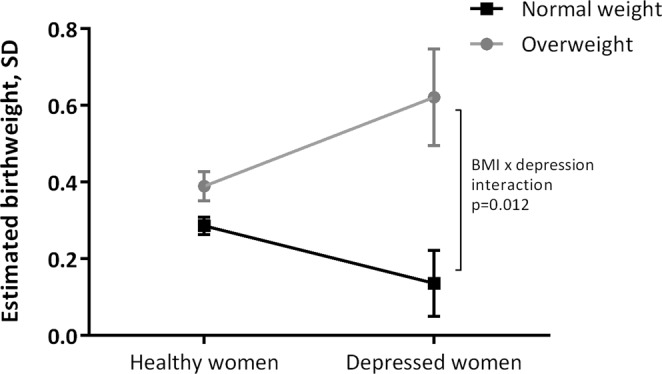
Interaction between maternal BMI and antenatal depression on standardized birthweight.

The interaction between antenatal depressive symptoms, maternal BMI and standardized birthweight was investigated by ANCOVA. Following adjustment for confounders a significant interaction between BMI and depression case-control status was noted, F(1,3840) = 6.82; p = 0.009, Fig. 1. This interaction remained unchanged when BMI, as a continuous variable, was entered as an additional covariable in the model, F(1,3839) = 6.32; p = 0.012. The interaction was primarily driven by depressed women with BMI 25 or more who gave birth to infants with greater birthweight than non-depressed overweight women, whereas the opposite pattern was noted in normal weight women, Table 4 (Model 1). When using LGA as outcome, a significant interaction between antenatal depressive symptoms and maternal BMI was also noted; AOR 3.73, 95% CI (1.62–8.51). However, this interaction did not remain following adjustment for BMI as a continuous variable; AOR 2.09, 95% CI (0.98–4.98).

**Table 4:** Multivariable linear regression analysis on the interaction between maternal BMI, antenatal depression and birthweight (SDS) in the primary study population. Model 1 adjusted for known confounding factors, Model 2 adjusted also for gestational weight gain as a potential mediator of the relationship.

Model 1^a^ Variable	Unstandardized β (95% CI)	β	*P*
Normal BMI, comorbid depressive symptoms	−0.14 (−0.31–0.30)	−0.03	0.106
Overweight, non-depressive	0.32 (0.25–0.38)	0.15	<0.001
Overweight, comorbid depressive symptoms	0.59 (0.34–0.83)	0.07	<0.001
**Model 2** ^**a**^ **Variable**	**Unstandardized β (95% CI)**	**β**	*P*
Normal BMI, comorbid depressive symptoms	−0.15 (−0.32–0.02)	−0.03	0.094
Overweight, non-depressive	0.35 (0.28–0.42)	0.17	<0.001
Overweight, comorbid depressive symptoms	0.64 (0.40–0.88)	0.08	<0.001
Weight gain gw 32	0.04 (0.04–0.05)	0.20	<0.001

^a^Adjusted for age, parity, height, smoking, country of origin, and educational level. gw = gestational week, CI = confidence intervals.

### The effect on birthweight is not mediated by greater weight gain in depressed women

Self-rated depression scores in gestational week 17 and 32 were in weak, but positive, correlation with maternal weight gain, both at gestational week 32 and 36, Table 3. The interaction between antenatal depressive symptoms, maternal weight gain (dichotomized at the median) and standardized birthweight was investigated by ANCOVA. Following adjustment for confounders a significant main effect of weight gain and birthweight was noted F(1,3613) = 19.62; p < 0.001, however, we found no interaction between weight gain and depression case-control status, F(1,3613) = 0.032; p = 0.857.

Further, when weight gain was entered in the linear regression analysis on BMI and depressive symptoms’ influence on standardized birthweight, the estimates were only marginally changed, Table 4 (Model 2). Thus, we pursued no path analyses on the mediating effect of weight gain.

## Discussion

Our results suggest that women who are both overweight and have symptoms of moderate to severe depression during pregnancy give birth to infants with greater birthweight than non-depressed overweight women. The opposite pattern was noted in normal weight women, although the difference between depressed and healthy normal-weight women was not significant.

Most previous studies on depression and birthweight have suggested an association with lower birth weight^6^, although there are some exceptions. For instance, Andersson *et al*. found a borderline-significant association between antenatal depressive disorder and birthweight of 4000 grams or more, although the association was lost when adjusted for maternal BMI^18^. Similarly, results from a large Canadian study suggested that the prevalence of macrosomia (>4500 g) and LGA infants were highest among the overweight or obese women with comorbid depression^19^. The significant interaction between depressive symptoms and maternal BMI noted in our study may help explain the discrepancies between our and prior results. First, as many other studies we find lower birth weight in the women who were classified as depressed, but importantly, no difference in standardized birthweight. Secondly, according to the interaction analysis, overweight women with antenatal depressive symptoms tend to give birth to children with higher birthweight, whereas the opposite pattern was seen in normal weight women with antenatal depressive symptoms, who instead tended to give birth to children with lower birthweight. Potentially, in populations with a greater proportion of normal weight or underweight women, the tendency towards lower birthweight, as noted in this study, is amplified. This is in line with the meta-analysis by Grote and co-workers where the association between antenatal depression and low birthweight was stronger in studies including women from developing countries or women with low socioeconomic status in the U.S^6^. The low birthweight noted in some studies may be the result of undeveloped or unequal maternal health care, whereas optimized maternal surveillance is able to counteract the depressogenic effects leading to low birthweight. Also, previous studies have not always addressed birthweight in relation to gestational length, why low birthweight simply may be the result of a depression-induced increased prevalence of preterm birth^6^. Finally, risk factors for low birthweight, such as smoking and low educational level, were less prevalent in our women than in the general pregnant population. Thus, the study population may be selected towards socioeconomic factors that drive the association in the opposite direction.

The increased birthweight in women with co-prevalent overweight and depressive symptoms was not explained by increased weight gain. Thus, the mechanism behind the influence of maternal BMI and antenatal depression on birthweight can only be speculated upon. Fetal growth is directly related to maternal nutrient availability and the ability of the placenta to transport these nutrients from maternal circulation to the fetus. In order to sustain appropriate fetal development the mother has to provide glucose, amino acids, and fatty acids to the fetus via the placenta^25^. Normal pregnancy is associated with insulin resistance and enhanced maternal hepatic glucose production^26^, both of which lead to increased glucose shunting to the fetus, fetal hyperinsulinemia, and enhanced fetal growth. We have recently demonstrated that placental gene expression in LGA infants born to non-diabetic mothers affects networks important for lipid metabolism, with insulin-like growth factor binding protein 1 being one of the top up-regulated genes, and leptin being a top down-regulated gene^27^. Leptin levels are as of yet unstudied in antenatal depression, but leptin levels at delivery are negatively associated with self-reported postpartum depressive symptoms^28^. Further, in non-pregnant women many factors, both peripheral and central, concerned with cell-mediated immunity, inflammation and oxidative and nitrosative stress, are related to the onset of depressive symptomes^29^. While the information on inflammatory response in women with antenatal depression is limited and inconsistent thus far^30^, we have recently provided evidence for an misadaptive switch to the anti-inflammatory milieu of the third trimester in women with antenatal depression^31^. Finally, the hypothalamus-pituitary-adrenal (HPA) axis is yet another potential link between maternal overweight, antenatal depression and neonatal complications^32^. In depressed pregnant women the HPA alterations seem to be regulated differently than in healthy pregnant women^32–36^. We and others have demonstrated that the cortisol to cortisone ratio, potentially a marker of maternal and placental 11β-hydroxy steroid dehydrogenase 2 activity, is positively associated with birthweight in women with psychiatric morbidity^36,37^. By this mechanism, psychiatric morbidity seems to increase fetal exposure to cortisol, which ultimately will influence fetal growth.

Our study has a number of limitations that should be mentioned. First, the study population consists of women with higher education level and lower rate of non-Nordic women than the general population, and therefore can we assume that our study group has a higher socio-economic status than average, and a healthy selection bias. However, for the outcomes of interest we note a numerically similar birthweight and a similar prevalence of LGA infants as in the general population. We used EPDS, a self-reported screening for antenatal depression, and not a psychiatric interview for establishment of depression diagnoses based on DSM-5 criteria. We tried to compensate for this by using to cut-off of 17 or more in the EPDS for our cases, which yielded a prevalence of antenatal depression within the range reported in the literature^38^. Further, given the established relationship between anxiety and birthweight, and especially comorbid anxiety and depression influence on birth weight, further insight could have been gained if antenatal anxiety had been captured in this study as well.

In conclusion, our study has revealed that first trimester maternal body mass index seems to modulate the association between antenatal depressive symptoms and birthweight. It remains to determine if our results can be conveyed to other populations or settings, and further insight is needed on the potential mechanisms underlying our findings. Nevertheless, this result emphasizes the importance of prevention, diagnosis and treatment of women with overweight and depression during pregnancy to minimize the complications for both the mother and the infant. Mental health problems are common comorbidities in obese women, and clinicians should actively ask about depressive and anxiety symptoms at the first antenatal booking. Further, these women should be counselled on physical activity and life style interventions during pregnancy, as this may limit the adverse outcomes for both mother and child.

## Supplementary information

Supplementary table

## Data Availability

The datasets generated during and/or analysed during the current study are available from the corresponding author on reasonable request.
