# Supplementary material for: Maternal body mass index moderates antenatal depression effects on infant birthweight
Source: Sci Rep. 2019 Apr 17;9:6213. doi: 10.1038/s41598-019-42360-1 (PMC6470129; doi:10.1038/s41598-019-42360-1)
Supplement: Supplementary file 1 — Supplementary table [file 41598_2019_42360_MOESM1_ESM.docx]

Maternal body mass index moderates antenatal depression effects on infant birthweight

Heidrun Petursdottir Maack^1^, Alkistis Skalkidou^1^, Anna Sjöholm^1^, Karin Eurenius-Orre^1^, Ajlana Mulic-Lutvica^1^, Anna-Karin Wikström^1^, Inger Sundström Poromaa^1^.

**Supplementary table 1**. Birthweight in relation to categorical variables in the primary study population.

| Variable |  | Standardized birthweight scores  Mean ± SD | P |
| --- | --- | --- | --- |
| Parity | Nulliparous | 0.12 ± 0.92 | <0.001 |
|  | Multipara | 0.49 ± 0.97 |  |
| BMI | < 18.5 | -1.30 ± 0.85 | <0.001 |
|  | 18.5-24.9 | 0.22 ± 0.92 |  |
|  | 25– 29.9 | 0.51 ± 0.99 |  |
|  | 30 -34.9 | 0.49 ± 0.96 |  |
|  | >35 | 0.74 ± 1.28 |  |
| Smoking | Yes | 0.27 ± 1.04 | 0.7 |
|  | No | 0.32 ± 0.96 |  |
| Nordic origin | Yes | 0.33 ± 0.97 | 0.01 |
|  | No | 0.16 ± 0.87 |  |
| Educational level | > 12 years | 0.29 ± 0.95 | 0.04 |
|  | ≤ 12 years | 0.41 ± 1.01 |  |
| Civil status | Married/cohabiting | 0.32 ± 0.97 | 0.9 |
|  | Single | 0.29 ± 0.91 |  |
| Use of antidepressants | Yes | 0.23 ± 1.02 | 0.4 |
|  | No | 0.32 ± 0.96 |  |
|  | Before pregnancy | 0.41 ± 1.08 |  |
| Pre-pregnancy hypertension | Yes | 0.39 ± 1.01 | 0.8 |
|  | No | 0.32 ± 0.96 |  |
| Polycystic ovary syndrome | Yes | 0.32 ± 1.09 | 1.0 |
|  | No | 0.32 ± 0.96 |  |
| Rheumatoid or inflammatory disorder | Yes | 0.15 ± 0.98 | 0.2 |
|  | No | 0.32 ± 0.96 |  |

Depressed women are defined as women with EPDS score ≥ 16 in either gestational week 17 or 32.
